# Improved Goldstein Interferogram Filter Based on Local Fringe Frequency Estimation

**DOI:** 10.3390/s16111976

**Published:** 2016-11-23

**Authors:** Qingqing Feng, Huaping Xu, Zhefeng Wu, Yanan You, Wei Liu, Shiqi Ge

**Affiliations:** 1 School of Electronic and Information Engineering, Beihang University, Beijing 100191, China; 18010127731@163.com (Q.F.); wuzheming001@126.com (Z.W.); yyngc@126.com (Y.Y.); 2 Electronic and Electrical Engineering Department, University of Sheffield, Sheffield S1 3JD, UK; w.liu@sheffield.ac.uk; 3 Nanjing Research Institute of Electronics Technology, Nanjing 210039, China; raymond2464@126.com

**Keywords:** interferometric synthetic aperture radar (InSAR), Goldstein interferogram filter, local fringe frequency estimation

## Abstract

The quality of an interferogram, which is limited by various phase noise, will greatly affect the further processes of InSAR, such as phase unwrapping. Interferometric SAR (InSAR) geophysical measurements’, such as height or displacement, phase filtering is therefore an essential step. In this work, an improved Goldstein interferogram filter is proposed to suppress the phase noise while preserving the fringe edges. First, the proposed adaptive filter step, performed before frequency estimation, is employed to improve the estimation accuracy. Subsequently, to preserve the fringe characteristics, the estimated fringe frequency in each fixed filtering patch is removed from the original noisy phase. Then, the residual phase is smoothed based on the modified Goldstein filter with its parameter alpha dependent on both the coherence map and the residual phase frequency. Finally, the filtered residual phase and the removed fringe frequency are combined to generate the filtered interferogram, with the loss of signal minimized while reducing the noise level. The effectiveness of the proposed method is verified by experimental results based on both simulated and real data.

## 1. Introduction

As an all-weather all-time remote sensing technique, the synthetic aperture radar (SAR) is of great importance in many fields, such as natural hazards’ monitoring, ocean investigation, geographic mapping, and so on [1,2,3]. Synthetic aperture radar interferometry (InSAR), a further development of the traditional SAR technology, employs two or more SAR antennas to retrieve the height profile or the deformation of the ground surface [4,5,6]. Due to its high measurement accuracy, the InSAR technique has been applied in a wide range of areas, such as forest height estimation [7], electronic warfare [8], ocean measurements [9] and geolocation determination of ground targets [10]. The main InSAR processing procedures include image registration, interferogram generation, phase unwrapping, etc. [4,11]. In practice, the quality of the generated interferogram is limited by phase noise due to co-registration errors, thermal noise, temporal decorrelation, baseline decorrelation, electromagnetic interference, and so on [12,13]. To reduce the phase unwrapping difficulty and improve the precision of the unwrapped phase, phase filtering has become an essential step for InSAR data processing [14,15,16]. An ideal phase filter should be able to reduce phase residues significantly while preserving the fringe details well [17]. Since failure in edge preservation will cause serious errors in subsequent procedures, fringe preservation is also a very important topic.

In recent years, various filtering methods were proposed to improve the quality of the interferometric phase. The simplest ones are the mean filter in the spatial domain and the low-pass filter in the frequency domain. As an extension, in the spatial domain, the fringe adaptive smoothing approach, proposed by Lee et al. (commonly referred to as the Lee filter) [18], has been successfully applied for interferometric phase processing with direction-dependent windows. Another extension is the slope-compensated mean filter based on local fringe frequency estimation (topography adaptive filter) [19], and its effectiveness is greatly affected by the size and shape of the filtering window [19].

In the frequency domain, the coherent signal in the filtering patch accumulates and forms a dominant peak, while the power of uncorrelated noises disperses stochastically in different directions [20,21]. Based on this characteristic, many filtering algorithms have been further developed [14,22,23,24,25,26]. The traditional Goldstein filter, as a low-pass filtering method, smoothes the intensity of Fourier-transformed samples in overlapped interferogram patches [22]. It is widely used for InSAR because of its notable noise suppression capability and fast operation [14]. However, one disadvantage of this method is that it destroys phase continuity in those dense fringe regions. To keep more texture details in the interferogram, Baran et al. proposed an adaptive Goldstein filtering method with the parameter alpha varying according to the coherence of the filtering window [23]. To improve the estimation accuracy of alpha, Song et al. proposed modified Goldstein filters based on empirical mode decomposition and the adaptive-neighbourhood technique [24,25]. All of these modified versions have been shown to be more effective than the traditional Goldstein filter. However, although these methods can reduce most types of phase noise, their ability to preserve fringes and edges is limited. Since the filter response in each overlapping window can be essentially considered as a low-pass filter, the high frequency components of fringes are suppressed [26]. Therefore, these methods may result in loss of fine details in an interferogram, especially in areas with dense fringes and complex textures.

To address the aforementioned problem, in this work, an enhanced Goldstein filtering method is proposed to preserve the fringes by removing the local fringe frequency before phase filtering. Firstly, a mean-filter moving patch is utilized to prefilter the pixels before fringe frequency estimation. The size of the prefilter window is adjusted with the mean coherence value and the PSD (phase standard deviation) in each filtering patch. In this way, the areas with low coherence or high noise level are filtered with larger windows. Since the phase precision decreases sharply when the size of mean filtering approaches to or exceeds the critical averaging look numbers [27], the size of the prefiltering window is limited to prevent the distortion of fringes. Therefore, the subsequent frequency estimation, achieved by Fourier transforms, will be more efficient and accurate. Then, the detected fringe frequency in the estimation window is removed from the original noisy phase, and the remaining part is filtered by the modified Goldstein filter. The filtering parameter α, changing according to the mean coherence and the re-estimated residual phase frequency, not only prevents the areas of high coherence (less noise level) from being over-filtered, but also allows stronger filtering in low coherence (high noise level) regions. Finally, the local fringe frequency and the filtered residual phase are combined to generate the filtered interferogram. Compared to the original Goldstein interferogram filter, there are three changes proposed: prefilter with an adaptive window size, the application of fringe frequency estimation and the optimization of the parameter α. As shown in the simulation results, the new method can suppress noise more effectively while preserving the fringe well, even for fringes with strong curvatures.

The remainder of this paper is organized as follows. The improved Goldstein filter based on local frequency estimation is introduced in Section 2, and evaluation results based on the simulated dataset and collected real data are provided in Section 3. Conclusions are drawn in Section 4.

## 2. Improved Goldstein Filter Based on Local Frequency Estimation

### 2.1. Analysis of the Goldstein Filter

The Goldstein filter smooths the intensity of Fourier-transformed samples of small, overlapping interferogram patches. The spectrum of the filtered interferogram can be expressed as [22]:

(1) $$H\left(f_{x},f_{y}\right)=S{\left\{\left|Z\left(f_{x},f_{y}\right)\right|\right\}}^{\alpha }\cdot Z\left(f_{x},f_{y}\right)$$

where $f_{x}$ and $f_{y}$ respectively represent the spatial frequencies in range and azimuth, α is the filter parameter, $Z\left(f_{x},f_{y}\right)$ represents the Fourier spectrum of each filtering window and S{⋅} is a smoothing operator, which is normally achieved by a low-pass filter. The patches are defined as small windows of the interferogram, with overlaps to maintain continuity at the boundaries. The parameter α, taking a value in the range of [0, 1], indicates the desired effectiveness level of the filtering operation. For α=0, we have $H\left(f_{x},f_{y}\right)=Z\left(f_{x},f_{y}\right)$, which means no filtering applied. The filtering effect will become more significant with the increase of α. A large value of α will result in a loss of resolution in the filtered phases, while a small value will reduce the ability of noise suppression. In general, it is difficult to choose an appropriate value for α, and for the original Goldstein filter, α=0.5 is normally used to ensure a balance between noise suppression and phase preservation [24,25].

To find the best value for α, in [23], the relationship between α and the mean value of the absolute coherence is derived as:

(2) $$\alpha =1-\bar{\gamma }$$

where $\bar{\gamma }$ is the mean coherence value of the effective patch (patch minus overlap). Clearly, this choice prevents areas of high coherence from being over-filtered and meanwhile allows strong filtering in areas of low coherence, which can effectively reduce the loss of resolution in areas of high coherence.

### 2.2. Combination of Goldstein Filter and Local Frequency Estimation

The Goldstein filter discussed above has the following drawbacks. In each filtering window, the smoothing operator removes high frequency components of the noisy interferogram. As a result, the dense fringe, composed of high frequencies in the filtering window, is also removed. Therefore, the filtering process may destroy the fringe frequency and result in the loss of fine details, especially in areas of complex textures.

**Figure 1:**
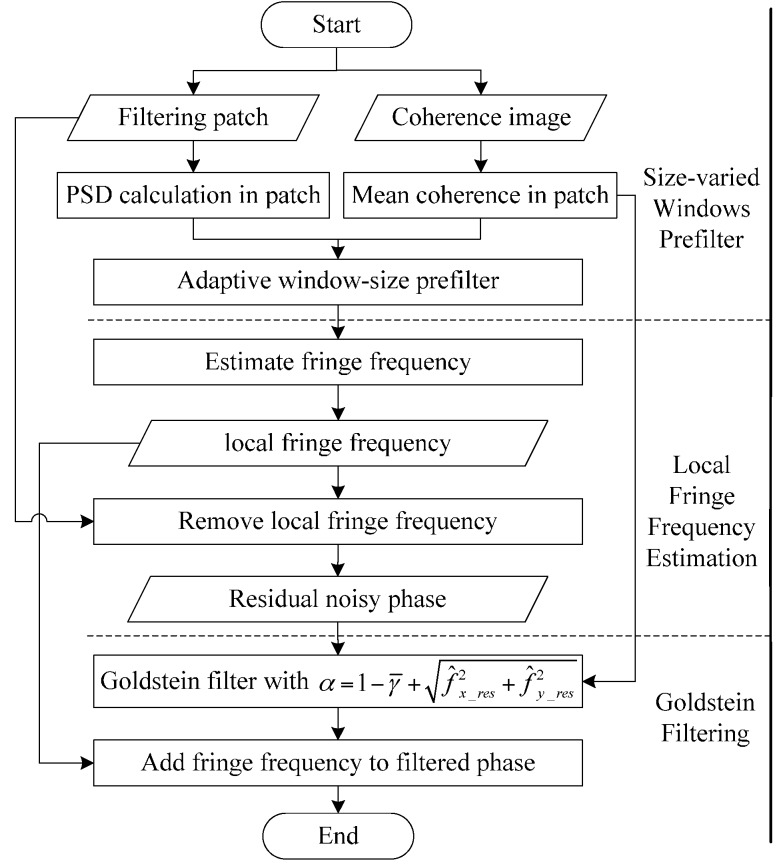
Flowchart of the improved Goldstein filtering method based on local frequency estimation. PSD, phase standard deviation.

To overcome this problem, we can incorporate the fringe frequency estimation technique into the Goldstein filter, which suppresses the noise of slope-compensated phase after estimating and removing the local fringe frequency in the filtering window. The overall flowchart for the improved method is shown in Figure 1, with the following three major steps:

1. The proposed adaptive mean filter is applied to ensure the accuracy of fringe frequency estimation. The prefilter window size, limited by the critical averaging look number, is varying according to the mean coherence value and PSD.
2. Fringe frequency estimation using Fourier transform is performed after adaptive mean prefiltering. Note that the estimated principal phase component is removed from the original noisy phase rather than the prefiltered phase. Hence, the prefiltering operation improves the accuracy of fringe frequency estimation and does not reduce the resolution of the interferogram.
3. The Goldstein filter is utilized to smooth the residual noisy phase with modified parameter α dependent on both the coherence map and residual phase frequency. The filtered residual phase and the removed fringe frequency are ultimately combined to derive the filtered interferogram.

There are three improvements to the original Goldstein filter: prefiltering with size-varied windows, estimating fringe frequency before the Goldstein filter and optimizing the Goldstein filter parameter α. In the following, we provide more details for each part.

#### 2.2.1. Size-Varied Windows Prefilter

Initially, to improve the accuracy of fringe frequency estimation, the prefiltering operation is implemented. To deal with phase noise, an adaptive mean filter is applied to prefilter the estimation patches. Thus, the complex interferogram of the centre pixel in the filtering window becomes:

(3) $$\bar{S}\left(x_{0},y_{0}\right)=\frac{1}{\left(2m+1\right)\left(2n+1\right)}\left(\sum_{x=x_{0}-m}^{x=x_{0}+m}\sum_{y=y_{0}-n}^{y=y_{0}+n}S\left(x,y\right)\right)$$

where S(x,y) denotes the original complex interferogram. The range and azimuth radius of the mean filtering patch are respectively limited by:

(4) $$m=\min\left\{\left\lfloor (1/\bar{\gamma })+\sigma \right\rfloor ,\;\left\lfloor (n_{r}-1)/2\right\rfloor \right\}$$

(5) $$n=\min\left\{\left\lfloor (1/\bar{\gamma })+\sigma \right\rfloor ,\;\left\lfloor (n_{a}-1)/2\right\rfloor \right\}$$

where min{⋅} takes the minimum value of its two parameters, $\bar{\gamma }$ is the mean coherence value in the estimation window, σ denotes the PSD in the estimation window and ⌊⋅⌋ rounds down its parameter to the nearest integer. $n_{r}$ and $n_{a}$ represent the critical averaging look numbers in the range and azimuth directions, respectively. The PSD is calculated by [25]:

(6) $$\sigma =\sqrt{\frac{\sum_{N}{\left(\varphi \left(x,y\right)-\bar{\varphi }\left(x,y\right)\right)}^{2}}{N-1}}$$

where φ(x,y) represents the phase value in the estimation window and $\bar{\varphi }\left(x,y\right)$ denotes the linear phase ramp in a moving patch. A larger PSD means a rough phase with more noise. In the range and azimuth directions, the critical averaging look numbers calculated by a priori information of the SAR imaging mode and control point altitude are respectively expressed as [27]:

(7) $$n_{r}=\left\lfloor \frac{\lambda r}{4BR_{r}}\cdot \frac{1}{\cos\beta /\sin(\theta +\beta )-\sin\theta }\right\rfloor \;$$

(8) $$n_{a}=\left\lfloor \frac{\lambda rr_{g}}{4B}\cdot \frac{1}{R_{a}^{2}\tan^{2}\beta +2R_{a}H\tan\beta }\right\rfloor$$

where λ is the radar operating wavelength, r is the slant range between the master antenna and the target on the ground, B is the length of baseline, $R_{r}$ is the slant range resolution, θ is the look angle, β represents the angle of ground inclination, $r_{g}$ is the projection of r on the ground and H denotes the satellite flight altitude.

As can be seen, the areas of lower coherence or more noise will be prefiltered with larger windows, while the low noise region will be prefiltered with smaller windows.

#### 2.2.2. Principal Phase Component Estimation

Next, fringe frequency estimation is performed after the above prefiltering operation. According to the model of the noisy phase described in [21], the power spectrum is characterized by a principal narrow-band component and a broadband component. The principal phase component corresponds to the fringe frequency of the real phase, while the broadband component includes the residual phase component and the phase noise. To derive the local fringe frequency of the filtering window, the maximum likelihood (ML) method [15] is employed using Fourier transforms [28,29,30]. The local frequency of a (2P+1)×(2Q+1) window in the interferogram can be expressed as:

(9) $$\left({\hat{f}}_{x},{\hat{f}}_{y}\right)=\arg\;\underset{f_{x},f_{y}}{\max}\left(\left|\sum_{x=x_{0}-P}^{x=x_{0}+P}\sum_{y=y_{0}-Q}^{y=y_{0}+Q}\bar{S}\left(x,y\right)\exp\left(-j2\pi \left(xf_{x}+yf_{y}\right)\right)\right|\right)$$

where $\bar{S}\left(x,y\right)$ represents the prefiltered complex interferogram and $\left(x_{0},y_{0}\right)$ denotes the centre pixel in the local fringe frequency estimation window. To speed up the optimization of Equation (9), FFT (fast Fourier transform) is usually employed. Then, the slope-compensated pixel in a (2P+1)×(2Q+1) patch can be obtained as:

(10) $$S^{\prime }\left(x,y\right)=S\left(x,y\right)\exp\left(-j2\pi \left(x{\hat{f}}_{x}+y{\hat{f}}_{y}\right)\right)$$

where $x\in \left[x_{0}-P,x_{0}+P\right]$, $y\in \left[y_{0}-Q,y_{0}+Q\right]$, S(x,y) represents the original noise phase before prefiltering and $S^{\prime }\left(x,y\right)$ denotes the slope-compensated complex phase. Since the estimated principal phase component is subtracted from the original noisy phase, the prefiltering operation improves the accuracy of fringe frequency estimation without reducing the resolution of the interferogram.

#### 2.2.3. Residual Noisy Phase Filter

In this part, the residual noisy phase, containing the noise and the residual phase component, is further discussed. In an ideal interferogram without noise, the residue phase without fringe frequency approaches zero in the frequency domain. Nevertheless, the frequencies of the residual phase are not close to zero in practice due to different types of phase noise. Therefore, a change to the Goldstein parameter α is required depending on both the residual phase frequency and coherence map. Firstly, the dominant frequency spectrum amplitude of the residual noisy phase in a (2P+1)×(2Q+1) window can be obtained as:

(11) $$\left({\hat{f}}_{x\_res},{\hat{f}}_{y\_res}\right)=\arg\;\underset{f_{x\_res},f_{x\_res}}{\max}\left(\left|\sum_{x=x_{0}-P}^{x=x_{0}+P}\sum_{y=y_{0}-Q}^{y=y_{0}+Q}S^{\prime }\left(x,y\right)\exp\left(-j2\pi \left(xf_{x\_res}+yf_{y\_res}\right)\right)\right|\right)$$

where $f_{x\_res}$ and $f_{y\_res}$ respectively represent the range and azimuth frequency component of the residual noisy phase in the (2P+1)×(2Q+1) window. In Equation (11), a larger frequency means greater noise intensity. Therefore, we can combine the dominant frequency spectrum amplitude and coherence value to modify the Goldstein filter parameter into:

(12) $$\alpha =1-\bar{\gamma }+\sqrt{{\left|{\hat{f}}_{x\_res}\right|}^{2}+{\left|{\hat{f}}_{y\_res}\right|}^{2}}$$

This modification ensures that the incoherent or noisy areas are filtered more than coherent or low-noise ones. The filtering operation is implemented by combining Equations (1) and (12). To express it more clearly, we define the filtered residual complex phase as ${\hat{S}}^{\prime }\left(i,j\right)$. Accordingly, the derived interferogram filtered by our method can be written as:

(13) $$\hat{\varphi }\left(i,j\right)=\arg\left\{{\hat{S}}^{\prime }\left(i,j\right)\exp\left(j2\pi \left(i{\hat{f}}_{i}+j{\hat{f}}_{j}\right)\right)\right\}$$

where (i,j) denotes the position in the interferogram. $\left({\hat{f}}_{i},{\hat{f}}_{j}\right)$ represents the estimated frequencies derived by Equation (9) in a local window centred on (i,j). In Equation (13), the processed interferometric phase is composed of two parts: the estimated fringe frequency and the filtered residual phase. This enhanced Goldstein filter will effectively prevent the local fringe frequency from being suppressed by the low-pass filter. Therefore, slope compensation can effectively reduce phase noise and at the same time maintain the fringe details.

**Figure 2:**
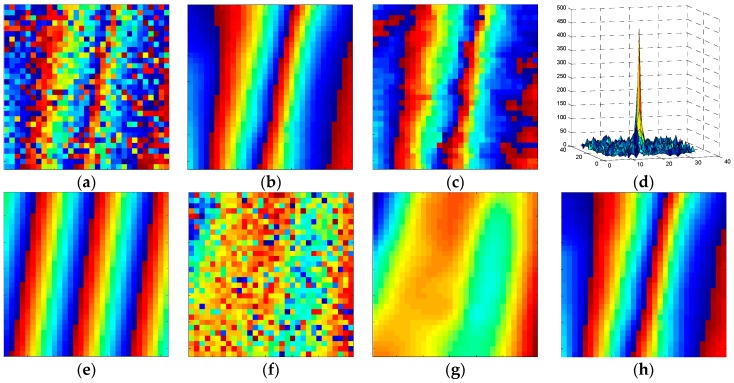
The example for the procedure of our method by using a window with 31 × 31 pixels. (**a**) Noisy phase window; (**b**) corresponding simulated true phase; (**c**) phase after prefiltering; (**d**) principle power spectral density of the prefiltered phase; (**e**) the removed principal phase component; (**f**) residual noisy phase; (**g**) filtered residual phase; (**h**) final processed phase patch by our method.

Figure 2 shows the detailed procedure of our method with a patch of 31 × 31 pixels. The original noisy patch and the corresponding real phase are respectively presented in Figure 2a,b. Then, the phase after prefiltering is obtained, as shown in Figure 2c. Subsequently, the dominant frequency spectrum amplitude is derived in Figure 2d, and the local fringe component is produced in Figure 2e. By subtracting the local fringe component from the original noisy phase, the residual phase is obtained as Figure 2f. The residual noisy phases, composed of residual phase components and phase noise, are further filtered by using Equations (1) and (12), as shown in Figure 2g. Finally, the local fringe component and filtered residual phase constitute the filtered interferogram (Figure 2h), which is closer to the real phase (Figure 2b). As can be seen, after these modifications, texture details of the effective phases are preserved while the regions with strong noise are filtered more, which provides a more accurate filter for the interferogram.

## 3. Results and Analysis

In this section, the performance of the proposed method is studied using simulated and real data. The effect of the three proposed modifications to the standard Goldstein filter is demonstrated by simulated data; both the simulated and the real dataset are used to show the effectiveness of the proposed method in phase noise reduction and fringe preservation in comparison with several existing methods.

### 3.1. Comparison with Our Modifications

**Figure 3:**
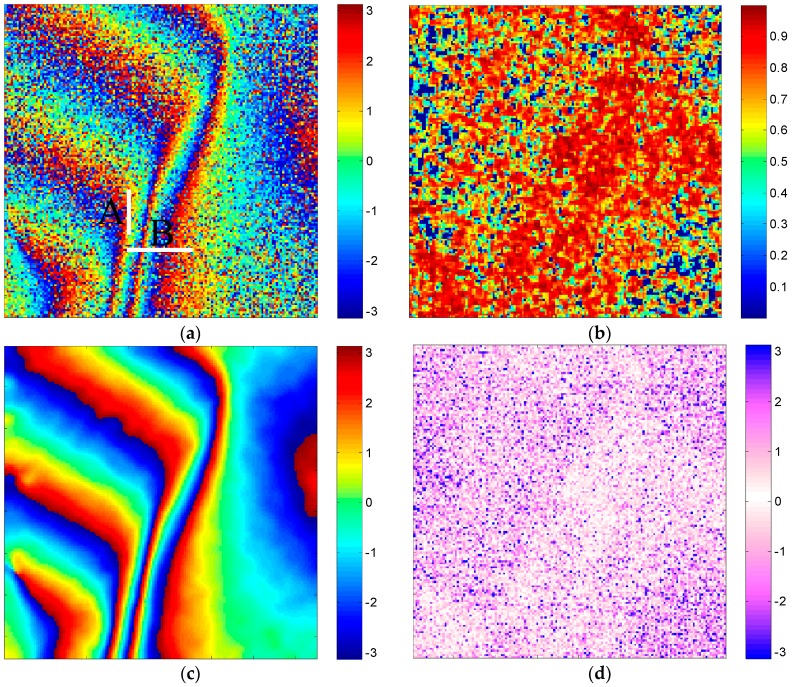
Simulated data. (**a**) Simulated noisy phase (Cross-sections A and B, respectively representing the transitional region in azimuth and the phase jumping region in range, will be further analysed in Section 3.2); (**b**) coherence map; (**c**) simulated true phase; (**d**) phase error image.

The interferogram is derived from the simulated complex SAR image pairs through interferometry. The SAR images with 150 × 150 samples are initially simulated according to the SAR geometry and the imaging area [31]. Subsequently, image co-registration is performed to derive the noisy interferogram, as shown in Figure 3a. The corresponding coherence map calculated by the coherence estimator [32] with 3 × 3 windows is presented in Figure 3b. For comparison, the true phase of the mountainous terrain is given in Figure 3c using the method in [33]. The corresponding phase error of the original noisy interferogram is shown in Figure 3d.

**Figure 4:**
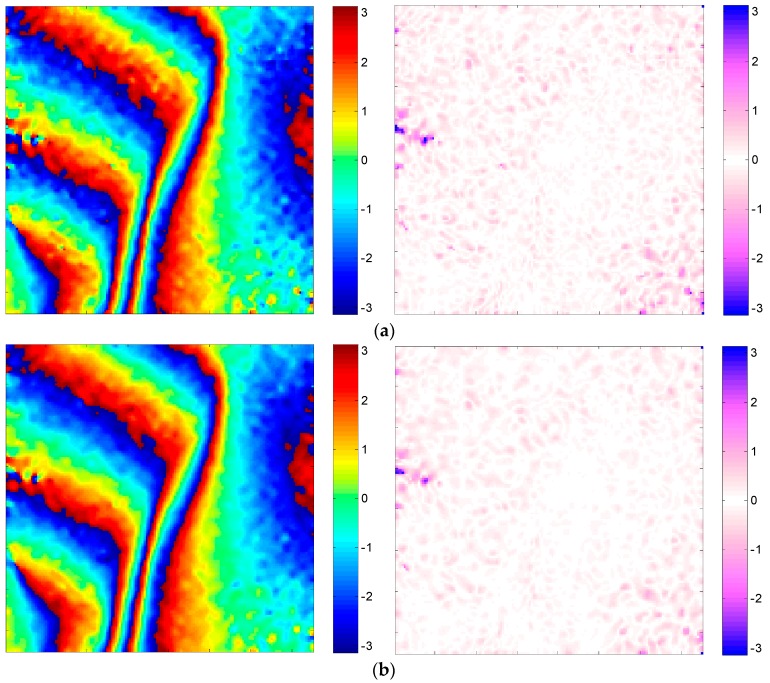

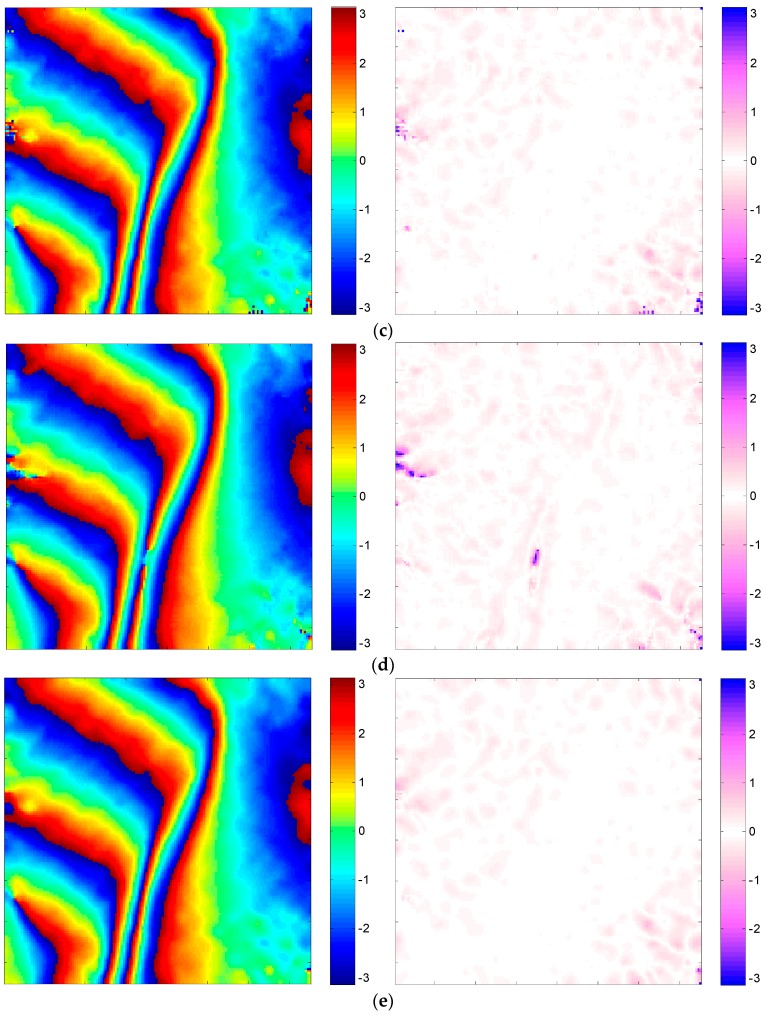
Simulated interferograms and corresponding error images using different modifications. (**a**) Reference Goldstein filter; (**b**) improved filter with Modification 1 only; (**c**) improved filter with Modification 2 only; (**d**) improved filter with Modification 3 only; (**e**) our method.

We filter the simulated data using five algorithms: the reference Goldstein interferogram filter [23], the improved filter with Modification 1 only (adding the adaptive prefilter to the reference Goldstein filter), the improved filter with Modification 2 only (removing fringe frequency before applying the reference Goldstein filter), the improved filter with Modification 3 only (with the Goldstein filter parameter α dependent on coherence and noise frequency) and the proposed method. The filtered results and the corresponding error images are respectively shown in Figure 4a–e. All of these methods use an 11 × 11 window for Goldstein phase noise filtering. Comparing the proposed Modification 1 with the reference Goldstein filter, as shown in Figure 4a,b, the residues are dramatically reduced due to the noise suppression capability of the adaptive mean prefiltering operation, leading to a more accurate fringe frequency estimation result based on Figure 4b. Figure 4c indicates that Modification 2 has a great advantage of preserving fringe continuity. The proposed Modification 3 makes Goldstein filter parameter α dependent on the absolute value of noise frequency, as well as coherence, as shown in Figure 4d, where although several dense fringes are not preserved well, such a modification has effectively increased the smoothness of the filtering result. The proposed method, combining the advantages of Modification 1, Modification 2 and Modification 3 (see Figure 4e), provides promising performance in both noise reduction and fringe preservation.

In order to evaluate the performance of our modifications in a quantitative way, the phase residue number, the edge preservation index (EPI) and the mean-square errors (MSE) between the filtered and the true phase are calculated. The number of residues determines the effectiveness of eliminating phase noise, and the EPI, indicating the capability of fringe and edge preservation, is given by [24]:

(14) $$EPI=\frac{\sum \left(\left|\varphi_{s}\left(i,j\right)-\varphi_{s}\left(i+1,j\right)\right|+\left|\varphi_{s}\left(i,j\right)-\varphi_{s}\left(i,j+1\right)\right|\right)}{\sum \left(\left|\varphi_{o}\left(i,j\right)-\varphi_{o}\left(i+1,j\right)\right|+\left|\varphi_{o}\left(i,j\right)-\varphi_{o}\left(i,j+1\right)\right|\right)}$$

where $\varphi_{o}\left(i,j\right)$ and $\varphi_{s}\left(i,j\right)$ represent the edge phase before and after filtering, respectively. In this experiment, $\varphi_{o}\left(i,j\right)$ is the true phase value. A better fringe means an EPI closer to one. Clearly, EPI>1 indicates more noise or false fringe details on the filtered interferogram, while EPI<1 means the fringe curvature of the filtered phase is decreased in comparison with the “true” phase. MSE, which is the mean-square error of the filtered phase, is defined as [15]:

(15) $$MSE=E\left[{\left|\arg\left(\exp\left(j\hat{\varphi }(i,j)-j\varphi_{real}(i,j)\right)\right)\right|}^{2}\right]$$

where E[⋅] denotes the statistics expectation, $\hat{\varphi }\left(i,j\right)$ represents the filtered InSAR phase and $\varphi_{real}\left(i,j\right)$ is the ideal phase value.

The filtered interferograms are evaluated using the aforementioned criteria. The results are shown in Table 1. It can be seen that Modification 1 is mainly for reducing the residues, while Modification 2 and Modification 3 have a good performance in fringe and edge preservation. Furthermore, all of those modifications have smaller MSE values than the reference Goldstein filter, which indicates an increased accuracy by the proposed method. These results clearly demonstrate that the proposed filtering method has a much better performance in reducing noise and preserving fringes than the reference Goldstein interferogram filter.

### 3.2. Comparison with Other Filters

**Figure 5:**
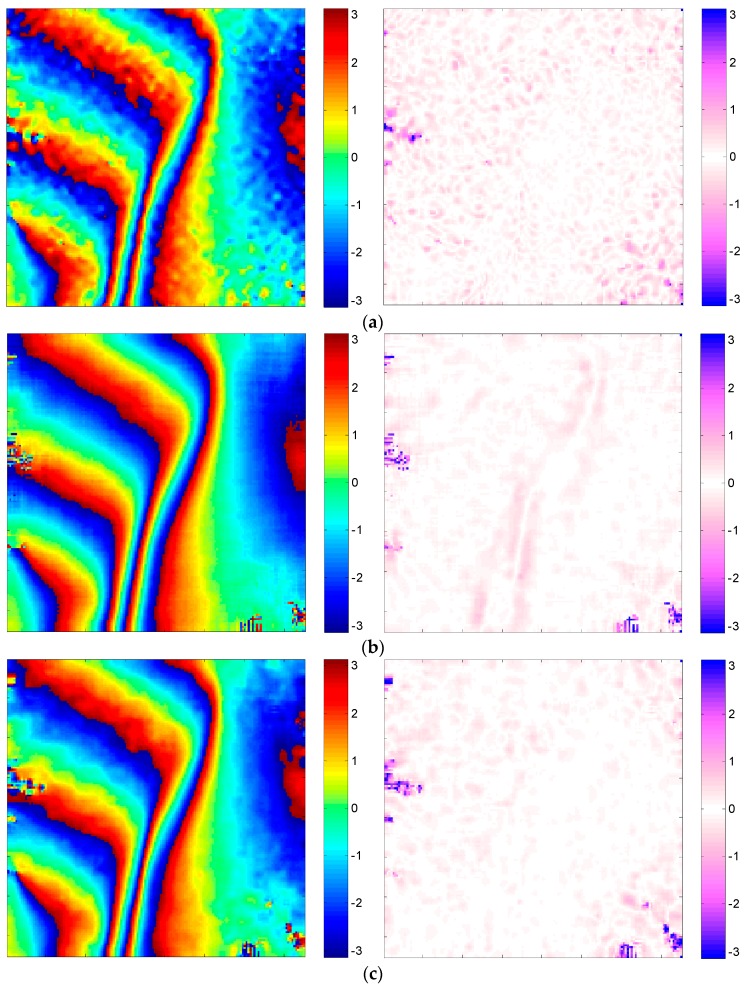

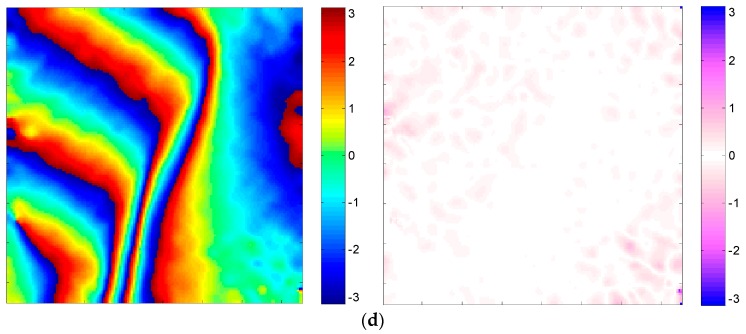
Filtered results and corresponding error images using different methods. (**a**) Reference Goldstein filter; (**b**) reference topography adaptive filter; (**c**) Lee filter; (**d**) our method.

In this part, the performance of our method is evaluated with the simulated dataset. As a comparison, the reference Goldstein filter [23], the topography adaptive filter [19] and the Lee filter [18] are also used, and the corresponding filtered results of different methods are shown in Figure 5a–c, where 11 × 11 phase denoising windows are used for all cases.

As shown in Figure 5a, the reference Goldstein filter is less capable of edge preservation in low-coherence regions compared with the true interferogram (Figure 3c). In Figure 5b, the reference topography adaptive filter induces phase errors along the fringe because of the inaccurate estimation of fringe frequency. In Figure 5c, several residues are introduced by the Lee filter, since a high noise level will cause serious problems for the orientation determination of the Lee filter, and a wrong window direction will result in an inaccurate filtered result. Figure 5d again demonstrates the superiority of the proposed method.

The calculated quantitative results are shown in Table 2. We can see that the filtered interferogram of the proposed method has the least residue number among these methods, indicating a very high noise reduction result. Moreover, the EPI and MSE obtained by our method have almost reached those of the real phase. This quantitative evaluation has again verified that the proposed method can not only eliminate phase noise more effectively, but also preserve the fringe well.

Then, in order to validate the robustness of our method in filtering the transitional region and phase jumping region, two cross-sections (A in the azimuth direction and B in the range direction) where phase distortion often occurs are extracted from Figure 3a. By comparing the cross-sections, the advantage of our method can be revealed.

**Figure 6:**
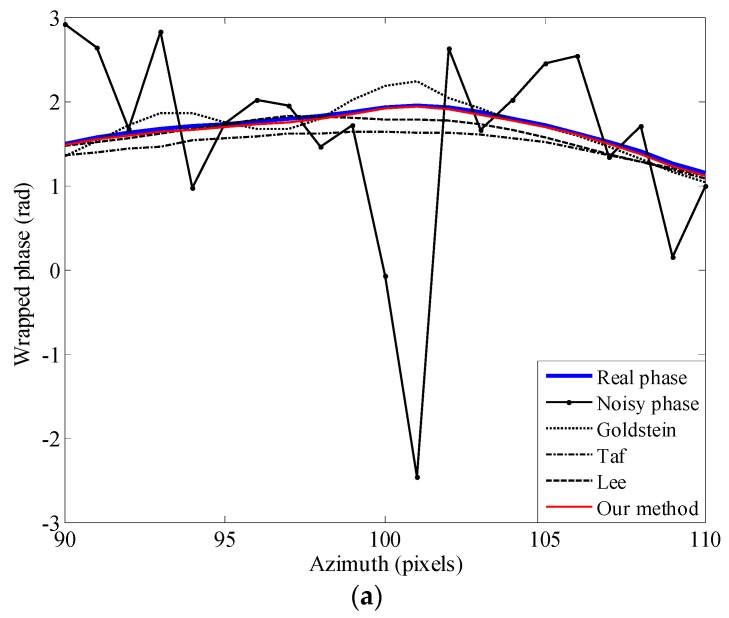

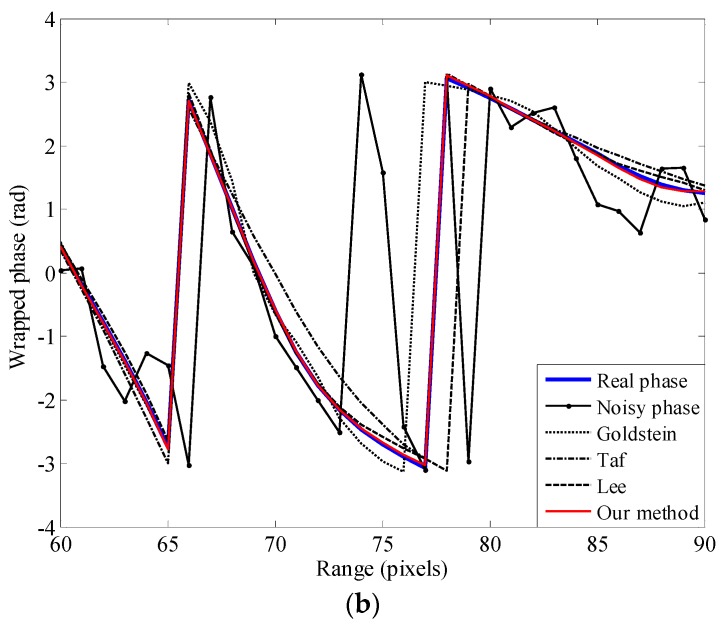
Cross-sections over the simulated interferogram, where “Taf” represents the “topography adaptive filter”. (**a**) Cross-section for A in the azimuth direction; (**b**) cross-section for B in the range direction.

As show in Figure 6a, the filtered phase obtained by our method is much closer to the real phase than others. In Figure 6b, the Goldstein filter and the Lee filter cannot achieve a very good result in the phase jumping area with a high level of noise and produce a shift in the fringe peak. In both figures, the topography adaptive filter leads to some variations in the transition zone. As can be seen, the proposed method has the best edge preservation performance among all four methods considered.

**Figure 7:**
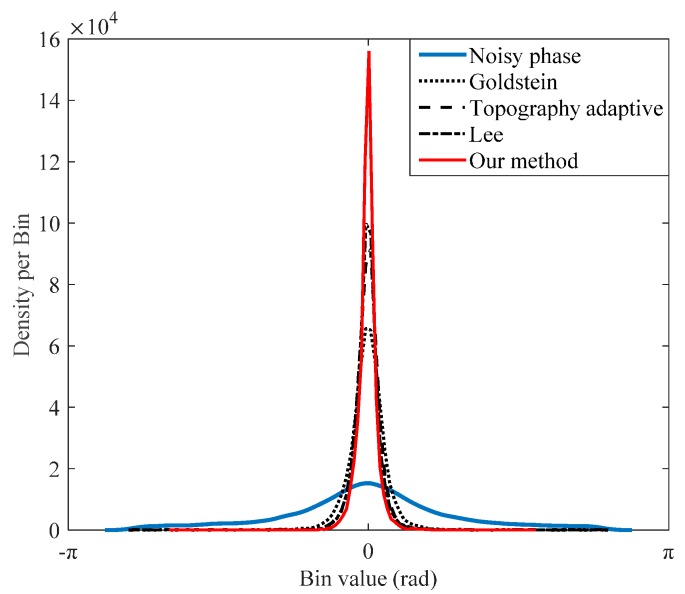
Density functions of phase error (filtered phase minus true phase). Phase error is wrapped to the range [−π,π).

Finally, the density function of phase errors (filtered phase minus the real phase) within the range [−π,π) is shown in Figure 7, and we can draw the same conclusion about our proposed method.

### 3.3. Real Data Experiment

In this part, the interferogram of the SIR C-SAR data (C-band, Etna Volcano of Italy) is used to investigate the performance of the proposed method. A typical area of the experimental results with a 400 × 400 sample dimension is selected.

**Figure 8:**
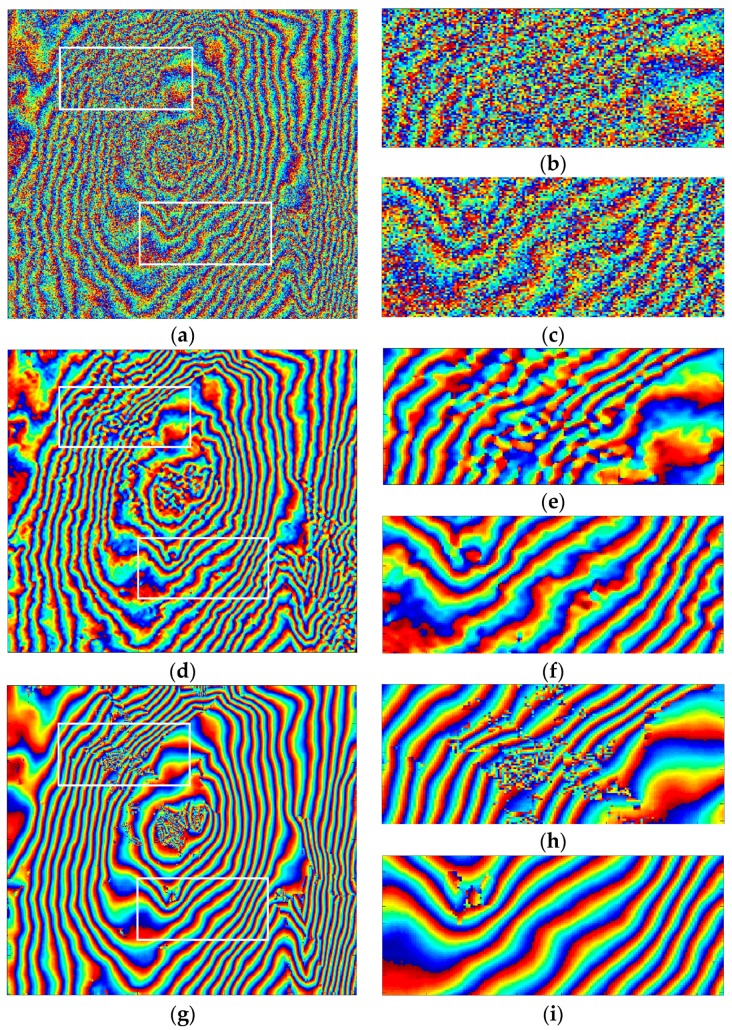

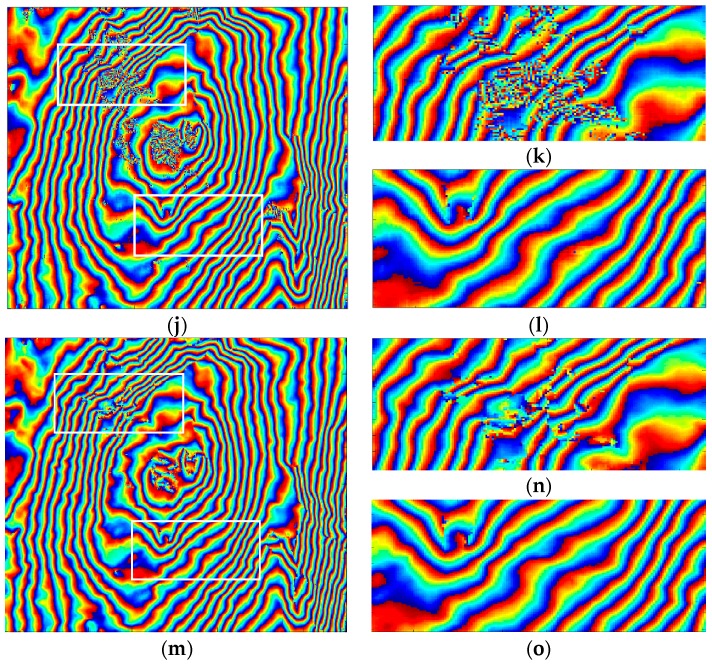
Filtered results using real data. The figures in the left column show the entire interferogram; the figures in the right column are enlarged areas corresponding to the white rectangles of the left column. (From left to right and top to bottom) (**a**–**c**) noisy interferogram; (**d**–**f**) reference Goldstein filter; (**g**–**i**) topography adaptive filter; (**j**–**l**) Lee filter; (**m**–**o**) proposed method.

In Figure 8, the entire phase image is presented in the left column, and the enlarged area in the white rectangle is on the right. The original noisy image is provided in Figure 8a–c. The fringes in Figure 8b, buried in significant noise in the white rectangle, represent the steep terrain of Mount Etna. As shown in Figure 8c, the enlarged area contains more texture details. The filtering results produced by the reference Goldstein filter, the topography adaptive filter, the Lee filter and our method with a constant denoising window size of 17 × 17 are shown in Figure 8d–o. As can be seen in Figure 8e,f, the reference Goldstein filter has reduced the phase noise effectively, but the fringes are ambiguous and not continuous in dense fringe areas. Comparing Figure 8g–i with Figure 8d–f, we see that the topography adaptive filter is more capable of fringe preservation than the reference Goldstein filter in dense fringe areas. However, the estimated fringe frequency by the topography adaptive method is not accurate, and the fringe details of the resultant interferogram are lost. In Figure 8j–l, the Lee filter shows a better performance in detail preservation than the reference Goldstein filter and topography adaptive filter. Nevertheless, its windows’ direction is difficult to determine, especially in areas with a high noise level, leading to broken fringes in some regions. Figure 8m–o shows the filtering result using the proposed method, which has effectively improved the accuracy of fringe frequency estimation and simultaneously smoothed noise. In the enlarged areas of Figure 8, the proposed method gives the best result in preserving texture details and has the most continuous fringe in the steep terrain.

Table 3 lists the results for phase residue number and phase standard deviation. In terms of residues in the interferogram, the improvements by the reference Goldstein filter, the topography adaptive filter and the Lee filter are 97.41%, 96.17% and 93.98%, respectively, while our proposed one is 99.05%, which is the best result. Besides, the PSD for the proposed method is the smallest, giving the best smoothing effect.

Overall, we can conclude that the proposed interferogram filter based on local frequency estimation has consistently outperformed the existing ones.

## 4. Conclusions

In this paper, a modified Goldstein filtering method to reduce the phase noise has been proposed. As demonstrated by experimental results based on both simulated data and real data, it can suppress noise effectively while preserving the fringe details well. In detail, we have the following four major findings:

- The adaptive prefiltering operation based on phase standard deviation and coherence can effectively improve the accuracy of local fringe frequency estimation for areas incoherent or with a high level of noise without reducing the resolution of the interferogram.
- The fringe frequency estimation and slope compensation before applying the Goldstein filter can significantly enhance its performance in edge preservation.
- The modified Goldstein parameter α, varying with coherence and the dominant frequency component in the residual noise phase, provides a promising result in noise reduction.
- Fringe frequency compensation and residual phase filtering are combined to reduce the number of phase residues significantly while preserving the fringe details well, even for fringes with strong curvatures.

As a result, the filtered interferogram can keep its fringe frequency components and also benefit from the reduced noise level provided by the Goldstein filter, leading to an improved performance by the proposed method.

## Figures and Tables

**Table 1:** Evaluation of three modifications (simulated data). EPI, edge preservation index.

Interferogram	Residues	EPI	MSE
Real phase	0	1	0
Noisy phase	3270	7.8684	1.3054
Reference Goldstein	14	1.3739	0.0707
Modification 1	5	1.2275	0.0461
Modification 2	20	1.0921	0.0295
Modification 3	15	1.0725	0.0447
Our method	2	1.0362	0.0171

**Table 2:** Evaluation of different filters (simulated data).

Interferogram	Residues	EPI	MSE
Real phase	0	1	0
Noisy phase	3270	7.8684	1.3054
Reference Goldstein	14	1.3739	0.0707
Topography adaptive	73	1.1515	0.0709
Lee filter	48	1.2059	0.0864
Our method	2	1.0362	0.0171

**Table 3:** Evaluation result of different filters (real data).

Interferogram	Residues		Phase Standard Deviation	
	Magnitude	Improvement	Magnitude	Improvement
Unfiltered	32,956	-	1.5968	-
Reference Goldstein	853	97.41%	0.8996	43.66%
Topography adaptive	1263	96.17%	0.9094	43.05%
Lee filter	1982	93.98%	0.9393	41.18%
Our method	313	99.05%	0.8903	44.24%
